# Cellular Pathogenesis of Hepatic Encephalopathy: An Update

**DOI:** 10.3390/biom13020396

**Published:** 2023-02-19

**Authors:** Kaihui Lu

**Affiliations:** 1 Institute of Biochemistry and Molecular Biology I, Medical Faculty, Heinrich Heine University Duesseldorf, 40225 Duesseldorf, Germany; kaihui.lu@uni-duesseldorf.de; Tel.: +49-211-81-05033; 2 Division of Cardiology, Pulmonology and Vascular Medicine, Medical Faculty, Heinrich Heine University Duesseldorf, 40225 Duesseldorf, Germany

**Keywords:** hepatic encephalopathy, hyperammonemia, manganese toxicity, oxidative/nitrosative stress, autophagy, mitochondria, inflammation, senescence, astrocyte

## Abstract

Hepatic encephalopathy (HE) is a neuropsychiatric syndrome derived from metabolic disorders due to various liver failures. Clinically, HE is characterized by hyperammonemia, EEG abnormalities, and different degrees of disturbance in sensory, motor, and cognitive functions. The molecular mechanism of HE has not been fully elucidated, although it is generally accepted that HE occurs under the influence of miscellaneous factors, especially the synergistic effect of toxin accumulation and severe metabolism disturbance. This review summarizes the recently discovered cellular mechanisms involved in the pathogenesis of HE. Among the existing hypotheses, ammonia poisoning and the subsequent oxidative/nitrosative stress remain the mainstream theories, and reducing blood ammonia is thus the main strategy for the treatment of HE. Other pathological mechanisms mainly include manganese toxicity, autophagy inhibition, mitochondrial damage, inflammation, and senescence, proposing new avenues for future therapeutic interventions.

## 1. Introduction

Hepatic encephalopathy (HE) is a syndrome of the central nervous system dysfunction caused by various liver diseases and/or portal shunts and is a common complication of liver diseases such as cirrhosis. The clinical manifestations of hepatic encephalopathy are multifaceted, including declines in the sensory, motor, and cognitive functions [1,2]. The most common forms of hepatic encephalopathy develop as a result of cirrhosis and chronic liver disease. They are usually grouped into two categories: minimal hepatic encephalopathy, which is clinically difficult to detect, and overt hepatic encephalopathy, in which symptoms are usually evident [3,4]. Hepatic encephalopathy can also be clinically classified into four levels according to the severity of symptoms, ranging from changes in behavior and consciousness (level 1), disorientation and tremors (level 2), confusion, incoherence, and sleep abnormalities (level 3) to, finally a comatose state (level 4) [5]. Although mild symptoms are usually reversible, cognitive impairment may not be completely reversed after hepatic encephalopathy subsides [6]. The prognosis for hepatic encephalopathy is poor, with more than half of cirrhosis patients diagnosed with overt hepatic encephalopathy living less than one year [2]. In most cases, the only curative treatment option for hepatic encephalopathy is liver transplantation, which improves the five-year survival rate to >70% in those able to undergo liver transplantation [2]. Therefore, a thorough and detailed understanding of HE pathogenesis is essential to improving the treatment options, considering the limitations of liver transplantation.

## 2. Pathophysiological Mechanisms of Hepatic Encephalopathy

Many relevant pathogenic factors (e.g., hyponatremia, inflammatory cytokines, benzodiazepines, and ammonia) have been proposed and experimentally validated for the pathogenesis of hepatic encephalopathy, but the molecular and cellular mechanisms involved in the pathogenesis of HE, especially those that impair astrocyte and neuronal function in the early stages of the disease, are still poorly understood [7]. It is generally believed that the pathophysiological basis of HE is hepatocyte necrosis, functional failure, and the accumulation of many toxic substances without hepatic detoxification. Those accumulated toxins enter circulation via naturally or surgically formed side branch shunts between the portal veins and cross the blood-brain barrier to the brain, thereby causing central nervous system dysfunction [8]. Although at the onset of hepatic encephalopathy, the metabolic disturbances in the body are multifaceted and the mechanisms are complex, key contributing factors are believed to be the elevation of blood toxins such as ammonia and the accumulation of inhibitory neurotransmitters caused by the disturbance of nitrogenous substance metabolism [8,9].

**Figure 1:**
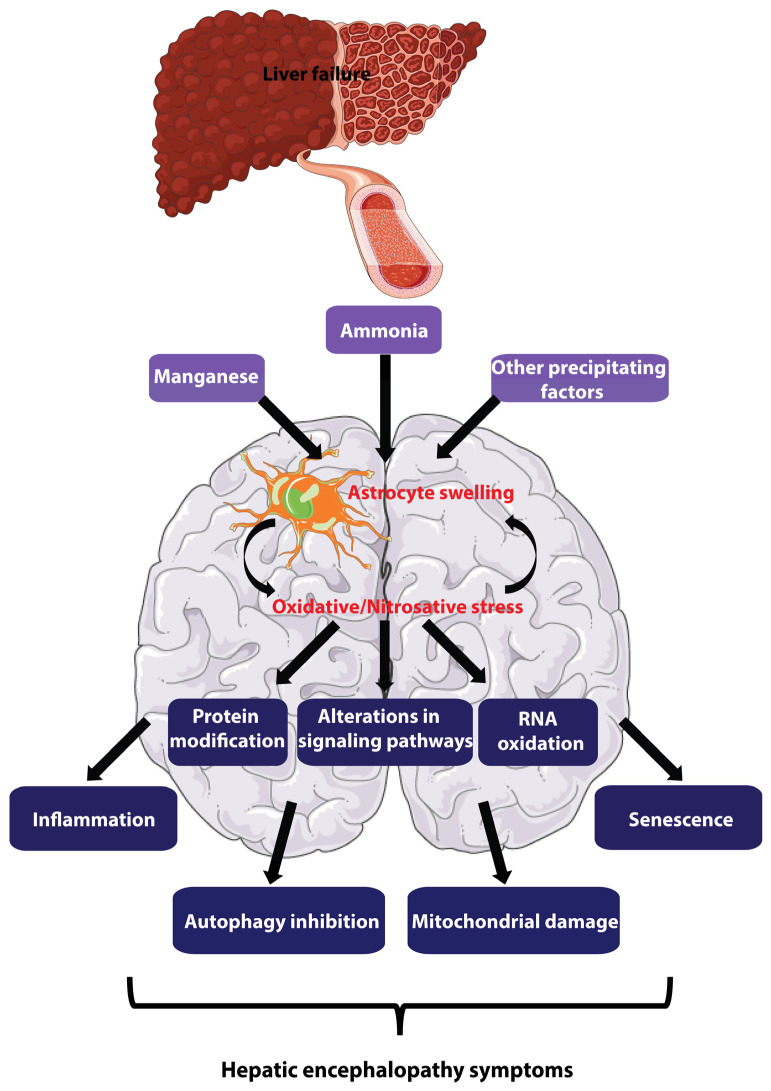
Major pathogenesis mechanisms of hepatic encephalopathy. Under liver dysfunction conditions, ammonia, manganese, and other heterogeneous precipitation factors cause astrocyte swelling and enhanced oxidative/nitrosative stress, which mutually reinforce each other. This self-amplifying loop then leads to a variety of harmful alterations in intracellular processes, including protein modification, RNA oxidation, multiple signaling pathway alterations, inflammation, autophagy inhibition, mitochondrial damage, and senescence. These unfavorable changes accumulatively impair astrocyte and neuron function/communication and stimulate the development of more severe HE symptoms. (Modified from [7,19]. The figure was partially generated using images from Servier Medical Art, licensed under a Creative Commons Attribution 3.0 Unported License).

Häussinger et al. first found that hepatic encephalopathy in patients with cirrhosis was caused by mild cerebral edema and oxidative/nitrosative stress in the brain [10,11]. In the brains of patients with cirrhosis and hepatic encephalopathy, levels of the organic osmolyte glutamine are elevated, while inositol levels are decreased [10]. The glutamine synthetase (GS) inhibitor methionine sulfoximine (MSO) completely prevents brain edema and ammonia-induced astrocyte swelling [12]. Since GS is primarily confined to astrocytes in the brain [13], this mild cerebral edema was thought to be the result of astrocyte volume regulation depletion caused by elevated blood ammonia, establishing the importance of astrocytes in the study of hepatic encephalopathy pathogenesis [7,14]. Subsequent studies demonstrated that the reciprocal stimulation between astrocyte swelling and oxidative/nitrosative stress triggered protein modifications and RNA oxidation, alterations in gene expression, and signaling pathways further causing senescence [15] (Figure 1). Notably, these changes found in cellular and animal models were also observed in post-mortem brain samples from patients suffering from cirrhosis and hepatic encephalopathy [11,16,17]. These changes are associated with altered concentrations of neurotransmitters and their metabolites as well as changes in cortical excitability in patients, which in turn lead to behavioral, perceptual, and motor deficits at the systemic level [18]. 

Currently, the prevailing animal and in vitro models of HE are still dominated by models of ammonia toxicity. The following sections and Figure 1 outline the recently discovered molecular and cellular mechanisms of hepatic encephalopathy.

### 2.1. Ammonia Toxicity

Blood ammonia was one of the first identified biomarkers of HE, and thus the most prevailing theory of HE pathogenesis is the ammonia toxicity theory [20,21]. However, blood ammonia levels alone are not sufficient to predict disease progression, as there is considerable overlap in blood ammonia concentrations in patients at different stages [22]. Despite the controversy, some studies have observed a good correlation between blood ammonia levels and HE severity [23,24,25]. 

Normally, blood ammonia is mainly derived from the processes of protein catabolism by intestinal bacteria and the breakdown of glutamine by intestinal or renal glutaminases. After fixation by the urea cycle, the liver can maintain blood ammonia at a certain level (35–50 μmol/L). However, when liver lesions and/or portal shunts are present, incomplete detoxification leads to a significant increase in blood ammonia concentration. Hypokalemia can also substantially increase blood ammonia concentrations by inhibiting the urea cycle [26]. Blood ammonia can diffuse freely (NH_3_ form) and also cross the blood-brain barrier via ammonia transporters, potassium channels, and cotransporters (NH_4_^+^ form), further leading to elevated brain ammonia [27,28].

Ammonia can in principle enter neurons and astrocytes, but astrocytes are considered to be the main target cells for the toxic effects of ammonia because they form a component of the blood-brain barrier and are well metabolized, initially taking up most of the ammonia and detoxifying it. High ammonia concentrations can damage many structures and functions of astrocytes, including induction of cell swelling, which leads to increased intracranial pressure, inflammation, abnormal mitochondrial permeability, disruption of oxidative stress homeostasis, interference with energy metabolism, and alteration of pH [29,30,31]. Notably, in astrocytes, ammonia reacts with glutamate to form glutamine, and both ammonia and glutamine induce mitochondrial permeability abnormalities. However, this ammonia toxicity-induced mitochondrial permeability abnormality is not significant in neuronal cells [32]. Nevertheless, high blood ammonia can impair neurotransmission processes, including brain dysfunction due to the impaired function of the solute carrier 38 (Slc38) family [20,33]. Recent studies have also found that inhibition of the egl-9 family hypoxia-inducible factor 3 (EGLN3) can effectively regulate the mitochondrial apoptotic pathway and reduce ammonia toxicity-induced apoptosis [34].

### 2.2. Manganese Toxicity

Elevated blood manganese levels and manganese deposition in the brain are important pathological features of hepatic encephalopathy in patients with cirrhosis, as they are often associated with cholestasis, which slows down the normal excretion of manganese. In cranial MRI, HE patients with elevated blood manganese levels often show frontal cortical atrophy and enhanced signal in the basal ganglia, especially in the pallidum [35,36,37]. Occupationally manganese-exposed workers also show symptoms similar to those of hepatic encephalopathy, but the associated symptoms disappear when blood manganese levels are reduced [38].

Being an important component of mitochondrial enzymes such as GS, manganese accumulates mostly in the mitochondria of astrocytes [39]. In astrocytes, manganese toxicity mainly induces oxidative and nitrosative stress, alters mitochondrial membrane potential, impairs mitochondrial function, and contributes to astrocyte swelling, inflammation, and brain edema [40,41]. High concentrations of manganese decrease GS activity and expression in astrocytes [42]. Upregulation of nuclear factor-erythroid 2 related factor 2 (Nrf2) inhibits manganese-induced cellular damage [43].

Manganese toxicity also interferes with communication between astrocytes and neurons at multiple levels, particularly by interfering with the glutamine-glutamate cycle (GGC) and affecting neuronal metabolism and neurotransmission [44]. Manganese toxicity causes cognitive and memory deficits in mice [45]. In rat astrocytes and animal models, manganese can downregulate glutamate aspartate transporter protein (GLAST) and glutamate transporter protein 1 (GLT-1), leading to abnormal behavioral and motor functions [46]. Chronic exposure to high manganese also downregulates GLAST and GLT-1 transporter protein expression in the brains of non-human primates [47]. Further studies revealed that high concentrations of manganese suppressed GLAST and GLT-1 expression through activation of the transcription factor Yin Yang 1 (YY1) [48,49]. In astrocyte-specific knockout YY1 mice, manganese-induced neurotoxicity was significantly reduced [50].

### 2.3. Oxidative/Nitrosative Stress

The fundamental role of oxidative/nitrosative stress in the pathogenesis of hepatic encephalopathy has been well confirmed by many studies in animal and in vitro models of HE [51,52,53,54,55,56,57,58,59,60,61,62,63]. Importantly, studies using brain tissues from patients with hepatic encephalopathy have shown that alternative markers of oxidative/nitrosative stress, such as protein tyrosine nitration, RNA oxidation, heat shock protein 27, and oxidative/nitrosative stress-related genes, are also upregulated in post-mortem brain samples [11,17]. In particular, GS nitration in astrocytes confirmed the presence of oxidative/nitrosative stress in the astrocytes of HE patients [11]. 

It was found that oxidative/nitrosative stress induced by pathogenic factors such as ammonia, benzodiazepines, pro-inflammatory cytokines, and hypo-osmolality was strongly associated with N-methyl-d-aspartate receptor (NMDAR)-dependent elevated intracellular calcium ion concentrations in astrocytes [52,61,62,64]. Vesicular glutamate release further elevates calcium ion concentrations [65], and elevated calcium ion concentrations in turn activate nitric oxide synthase (NOS) and NADPH oxidase (NOX)-dependent reactive nitrogen oxide species (RNOS) formation [61,64,66]. Two NOS isozymes have been identified to contribute to ammonia-induced nitric oxide (NO) production in astrocytes in vitro. Neuronal isozymes (nNOS) are thought to contribute to the rapid formation of NO [61,67], while prolonged exposure to ammonia also upregulates inducible nitric oxide synthase (iNOS) and promotes NO production [61,68]. El-Mlili et al. found that in vivo, chronic hyperammonemia treatment suppresses both the basal and stimulated activities of nNOS in response to NMDAR activation via phosphorylation [69]. NOX isozymes 2 and 4 contribute to ammonia-induced reactive oxygen species production. Astrocytes cultured in ammonia-containing or hypotonic cell culture media can be activated within minutes by phosphorylation of NOX2 by protein kinase Cζ [60]. The NOX4 protein also appears to be upregulated after 24 h of exposure to ammonia [16]. Interestingly, both NOS and NOX have been shown to contribute to ammonia-induced astrocyte swelling [66,68,70]. Mitochondria have been shown to play an important role in the generation and accumulation of ammonia-induced cellular ROS, which is associated with glutamine synthesis and hydrolysis [32,71,72].

In many models of hepatic encephalopathy, downregulation of the master regulator of antioxidant responses, Nrf2, sharply exacerbates ammonia toxicity [15,73]. Recent studies have found that by modulating the Nrf2/HO-1 signaling pathway, vitamin E, genistein flavonoids, resveratrol, leucovorin, taurine, sodium hydrogen sulfide, carvedilol, and South African drunken egg root extract can antagonize ammonia-induced oxidative/nitrosative stress and cytotoxicity in various HE models [73,74,75,76,77,78]. 

### 2.4. Positive Feedback of Astrocyte Swelling and Oxidative/Nitrosative Stress

Studies of in vitro models of hepatic encephalopathy have revealed the common properties of many inducers of hepatic encephalopathy that trigger astrocyte swelling as well as RNOS formation [62,70,79]. Since osmolarity changes and oxidative/nitrosative stress are interrelated, Häussinger et al. proposed that there is a positive feedback self-amplification effect between cell swelling and RNOS, which mutually enhance each other [19,73,80]. This amplification effect leads to many changes in astrocytes, such as RNA modification, protein tyrosine nitration, and senescence, which further impair astrocyte and neuronal function and communication, thus disrupting neural oscillations and cortical excitability, and finally triggering hepatic encephalopathy [61,71,81].

The interaction between astrocyte swelling and oxidative/nitrosative stress is also reflected in changes in the expression of osmotic transporter proteins [82]. In vitro studies on rat astrocytes have shown that swelling induced by HE-triggered factors is associated with downregulations in mRNA levels of sodium-dependent inositol transporter protein (SMIT) and taurine transporter protein (TAUT) [82,83]. Downregulation of SMIT mRNA has also been observed in the cerebral cortex of rats receiving acute or chronic hyperammonemia treatment [82]. This downregulation has been shown to be RNOS-dependent, as NADPH oxidase inhibitors alleviate both RNOS-induced swelling and the downregulation of SMIT and TAUT mRNA [66,70,82]. 

Rao et al. found that in astrocytes, ammonia enhances cellular water uptake through oxidative/nitrosative stress-dependent upregulation of the water channel protein AQ4 in the plasma membrane [84,85]. Nevertheless, the physiological relevance of these findings is controversial [86]. Although knockdown of AQ4 in mice prevents brain edema formation in a model of acute liver failure [72], AQ4 membrane polarization remains well-functioning in the brains of rats with acute or chronic hepatic encephalopathy [87], and the expression level of AQ4 in the brains of patients is still unclear. Although astrocytes have been identified as an important source of RNOS, further studies have shown that neurons, microglia, fibroblasts, and endothelial cells also contribute to RNOS production in hepatic encephalopathy [57,88,89,90].

Reactive oxygen species (ROS) generated outside the brain could already contribute to brain dysfunction in hepatic encephalopathy. Bosoi et al. showed that although the cellular origin of ROS in peripheral blood remains to be determined, arterial plasma levels of hydrogen peroxide were elevated in bile duct-ligated rats; removal of ROS from peripheral blood prevented brain edema, whereas increasing ROS levels triggered brain edema [91,92]. These findings suggest that ROS originating outside the brain and hyperammonemia caused by liver disease may trigger cerebral osmotic stress in a synergistic manner. Notably, levels of the oxidative stress surrogate marker nitrotyrosine were also dramatically elevated in the peripheral blood of HE patients [93,94]. Nucleus swelling may affect nuclear transport processes and gene transcription [95], and it remains to be determined whether oxidative/nitrosative stress also triggers nucleus swelling in animal models of hepatic encephalopathy and in patients.

### 2.5. Consequences of Oxidative/Nitrosative Stress

#### 2.5.1. Protein Post-Translational Modifications

Hepatic encephalopathy-related factor-induced oxidative/nitrosative stress triggers protein post-translational modifications in vitro, including protein tyrosine nitration [52,61,62,64,96], phosphorylation [61,79], glycosylation [97,98], carbonylation, and ubiquitination [99,100].

Protein phosphorylation and tyrosine nitration regulate protein function [101], and in astrocytes, ammonia induces the phosphorylation of a range of proteins, including mitogen-activated protein kinase p38MAPK, extracellular signal-regulated kinase (ERK) 1/2, and c-Jun N-terminal kinase (JNK) 1 and 2 [61,79]. Activation of these MAP kinases leads to downregulation of sodium-dependent glutamate/aspartate co-transport proteins, triggering late cellular swelling and inhibition of glutamate uptake [79].

In astrocytes exposed to hypotonicity, ammonia, diazepam, and proinflammatory cytokines, oxidative/nitrosative stress triggers nitration of tyrosine residues in a variety of proteins, including GS, ERK1, peripheral-type benzodiazepine receptor (PBR), and sodium-potassium-chloride cotransport protein (NKCC1) [52,61,62,64,96]. Increased tyrosine nitration of GS was proposed to be a main reason for the reduced GS activity in various brain regions of portal anastomosed rats [102,103]. The mechanism may be that Tyr336 nitration occurring in GS activity centers interferes with adenosine triphosphate binding [104]. Enhanced protein tyrosine nitration and a significant decrease in GS-specific activity were also found in the cerebral cortex of HE patients [11]. Interestingly, extracts from lipopolysaccharide (LPS)-treated rat brain reversed GS nitration and inactivation, suggesting that GS nitration is a mechanism that regulates GS activity under conditions of oxidative stress in the brain [105]. Further studies have shown that the enzymatic activity of nitrated GS could be restored in an acidic environment [104].

Enhancement in the tyrosine nitration of GS was also found in the brain and liver of LPS-intoxicated rats [64,106,107], suggesting that sepsis may further impair the detoxification of the brain and liver in cirrhotic patients, thereby exacerbating hepatic encephalopathy. In the presence of impaired hepatic detoxification capacity, skeletal muscle is thought to be an important site for detoxifying ammonia synthesis via glutamine [108]. Studies in rats with portal vein shunts suggest that hepatic dysfunction could even elevate skeletal muscle GS activity [109], but whether GS tyrosine nitration is also present in their skeletal muscle remains to be investigated.

In astrocytes, ammonia also induces nitration and carbonylation of NKCC1. Similar to NKCC1 phosphorylation, both modifications enhance the transport activity of NKCC1, and NKCC1 activation is thought to contribute to ammonia-induced astrocyte swelling [96].

#### 2.5.2. Nuclear Acid Oxidation

In addition, RNA oxidation is acknowledged to be a consequence of HE-related oxidative/nitrosative stress [53,59]. In astrocytes, oxidative/nitrosative stress triggers the oxidation of guanine in ribosomal and messenger RNAs in the brain to form 8-oxoguanosine [51,53,59]. Oxidation of ribosomal RNA impairs protein translation, and messenger RNA oxidation may lead to protein misfolding and RNA degradation [110]. This could explain the ammonia-induced decrease in GLAST mRNA and protein levels [53,111,112,113]. Significant RNA oxidation was also found in neuronal cytosolic RNA granules and brain synapses in animal models, which may affect neurotransmission [53]. Elevated levels of RNA oxidation were also found in post-mortem human brain tissue from cirrhotic patients with HE, but further studies are needed to elucidate the effects of RNA oxidation on human brain dysfunction [11]. Interestingly, the RNA quality control protein TROVE2 was also found to be significantly upregulated in several in vitro and animal models of HE [114]. Although currently no much evidence available, it is highly possible that RNOS could also result in unfavorable oxidative modifications of other nucleic acids such as DNA, tRNA and miRNA, which would further facilitate HE progression.

#### 2.5.3. Alterations in Signaling Pathways

Ammonia-induced osmotic stress and oxidative/nitrosative stress also cause alterations in the expression levels of several important genes in the brain, especially in astrocytes [30,57,67,115,116,117]. Nitric oxide-mediated release of zinc ions from the protein thiol-zinc cluster triggers a nuclear accumulation mechanism of the metal response element-binding transcription factor (MTF) 1/2 and the specificity protein (SP) 1, which further activates transcription of metallothionein (MT) 1/2 and PBR mRNA [67]. The in vivo relevance of the upregulation of metallothionein levels has also been demonstrated in post-mortem brain samples from patients with hepatic encephalopathy [17]. Although upregulation of MT1/2 is thought to counteract the toxic effects due to hypotonicity and ammonia-induced elevation of free zinc ion concentration, upregulation of PBR may enhance the synthesis of neurosteroids [67]. In addition, ammonia upregulates multidrug resistance protein 4 (MRP4) via RNOS-mediated activation of the peroxisome proliferator-activated receptor (PPAR) α. The MRP4 mediates the release of neurosteroids from astrocytes and may contribute to enhanced γ-aminobutyric acid (GABA)-ergic neurotransmission in hepatic encephalopathy [118,119,120]. MRP4 mRNA and protein are also significantly elevated in macroscopic specimens from patients with hepatic encephalopathy [118]. Neurosteroids are also ligands for the bile acid receptor TGR5, and activation of TGR5 by neurosteroids triggers ROS formation in astrocytes [121]. In ammonia-treated astrocytes and post-mortem brain tissue from HE patients, downregulation of TGR5 may help counteract neurosteroid- and TGR5-mediated ROS formation [121]. Ammonia also regulates miRNA expression in an oxidative stress-dependent manner, where downregulation of miR-326-3p can target heme oxygenase 1 (HO-1) and NOX4, whose upregulation can lead to proliferation inhibition and senescence of astrocytes [16,81]. Similar to ammonia, manganese can also promote neurosteroid production by activating astrocyte PBR [122].

Görg et al. analyzed the transcriptome of post-mortem brain tissue from patients with cirrhosis and hepatic encephalopathy; compared to controls without cirrhosis, more than 600 genes were found to exhibit an altered expression level, including genes related to oxidative stress, proliferation, and microglia activation [17]. Interestingly, changes in the expression of many genes, such as PPARα, may counteract the pro-inflammatory pathways in the brains of patients co-diagnosed with cirrhosis and hepatic encephalopathy [17].

### 2.6. Autophagy Inhibition and Lysosomal Damage

Free diffusion and reaction with protons of ammonia lead to the formation of ammonium in cells, resulting in a pH increase in low pH organelles (e.g., lysosomes, endosomes), which leads to functional damage and dysregulation of related cellular processes. At this moment, studies exploring the effects of ammonia toxicity on astrocyte damage from the perspective of autophagy regulation are still in the preliminary stage. Lu et al. showed that low concentrations of ammonia (1 mM) stimulated autophagic flux while high concentrations of ammonia (3–5 mM) inhibited autophagy in HE in vitro and in vivo models [123]. Accumulation of LC3-II, the lipid form of microtubule-associated protein 1A/1B light chain 3B (LC3B), has also been observed in brain samples from patients with hepatic encephalopathy [123]. The accumulation of p62 and LC3B in the substantia nigra was also found in a mouse model of HE induced by acute liver failure [124]. Interestingly, transglutaminase 2 (TGM2), a stress response gene favorable for the late steps of autophagy, was proven to be strongly upregulated in various HE models and in the brains of HE patients, indicating activation of a negative feedback defense mechanism to counteract the effects of hyperammonemia-induced autophagy inhibition [123]. Since autophagy is a component of cellular and metabolic homeostasis, altered autophagic flux may have synergistic effects with cell proliferation inhibition, senescence, inflammation, and mitochondrial dysfunction in the pathogenesis of hepatic encephalopathy [123]. Another study also specified that dysfunctional mitochondrial autophagy is associated with the pathogenesis of nonalcoholic fatty liver disease [125]. 

Ammonia-induced inhibition of autophagy was shown to be pH- and ROS-dependent and could be partially overcome by the administration of taurine in vivo [123]. The promotion of autophagic flux by taurine may also explain its ability to alleviate ammonia toxicity and reduce hyperammonemia-induced brain edema [81,126]. In addition to taurine, metformin also alleviates ammonia toxicity-induced cellular senescence by improving autophagy [127].

### 2.7. The “Trojan Horse” Theory of Glutamine, Mitochondrial Damage, and Altered Cellular Energy Metabolism

**Figure 2:**
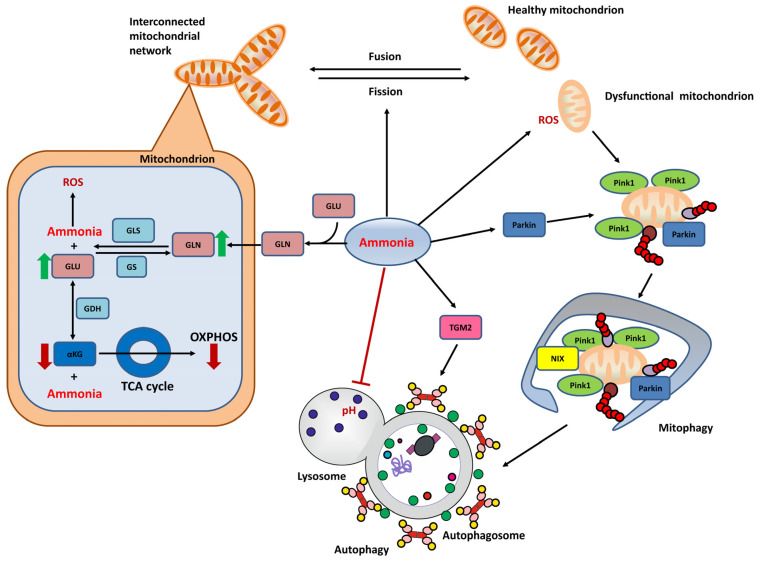
Major mitochondria-related pathogenesis mechanisms induced by ammonia toxicity in astrocytes. In the pathogenesis of HE, hyperammonemia directly affects mitochondrial function in astrocytes. One possible explanation is the “Trojan horse” hypothesis: transport of glutamine into mitochondria leads to the subsequent generation of ROS and ammonia inside mitochondria. Ammonia also leads to a dramatic increase in the synthesis of glutamine and glutamate and thus a partial depletion of αKG, which further decreases the capacity of oxidative phosphorylation. An increase in mitochondrial dysfunction naturally activates a variety of mQC processes. For mitochondrial dynamics, ammonia increases mitochondrial fission. For mQC degradation pathways, on the one hand, ammonia enhances mitophagy initiation, for example, via a ubiquitination-mediated pathway; on the other hand, ammonia blocks the late stages of autophagic degradation via pH alteration, which in turn promotes relevant defense mechanisms such as TGM2 upregulation. αKG:α-ketoglutarate; GDH:glutamate dehydrogenase; GLN:glutamine; GLS:glutaminase; GLU:glutamate; GS:glutamine synthetase; OXPHOS:oxidative phosphorylation; ROS:reactive oxygen species.

The “Trojan horse” theory describes the toxic effects of glutamine on mitochondria in astrocytes [128]. Briefly, glutamine accumulated in the cytoplasm enters the mitochondria and is converted to glutamate by glutaminase (GLS) I. Subsequently, glutamate is oxidatively deaminated to α-ketoglutarate (αKG) by glutamate dehydrogenase (GDH) to provide fuel and energy for the tricarboxylic acid (TCA) cycle. Both steps release free ammonia. Ammonia in the mitochondria alters the pH in the mitochondria and inhibits various mitochondrial enzymes and biochemical reactions, including the respiratory chain, thereby promoting RNOS production [58]. As a result of oxidative/nitrosative stress, altered mitochondrial permeability occurs [129], which is accompanied by mitochondrial fracture and swelling [71,130]. These mitochondrial abnormalities were observed not only in astrocytes but also in brain endothelial cells [131]. Damage to mitochondria often induces mitochondrial degradation, such as Parkin-mediated mitophagy [124]. However, as the downstream degradation is blocked, dysfunctional mitochondria are more likely to accumulate in cells [123,130]. These mitochondrial alterations affect astrocyte and neuronal function, triggering symptoms such as brain edema [132]. Indeed, both elevated glutamine and ammonia contribute to astrocyte swelling. Glutamine levels not only correlate with the severity of edema [28], but the use of the GS inhibitor MSO inhibits astrocyte swelling and reduces brain edema in rats with portal shunts [12,133]. Inhibition of glutamine transport to mitochondria ameliorates brain edema in an animal model of hepatic encephalopathy [134]. Figure 2 summarizes the mitochondria-related HE pathogenesis mechanisms in astrocytes.

The damage to mitochondria in neuronal cells due to manganese toxicity has also gained attention in recent years. After entering neuronal cells, manganese accumulates in the mitochondria mainly as Mn^2+^, Mn^3+^, and Mn^4+^. It was found that in nigrostriatal and striatal neuronal cells, low-valent manganese is converted to high-valent manganese. During this process, a dramatic increase of mitochondrial ROS and the opening of mitochondrial permeability transition pores lead to the inhibition of most enzyme complex activities along the electron transport chain, thus affecting energy synthesis [135,136]. 

In hepatic encephalopathy, the accumulation of toxins such as ammonia and manganese can significantly devastate mitochondrial function in brain cells, leading to multiple alterations in the energy and metabolic state of the brain. Reduced ATP levels have been found in ammonia-treated cellular models, acute or chronic hyperammonemic animal models, and acute liver failure patient samples [137,138,139]. A shift in brain cell energy metabolism from aerobic to anaerobic was found in acute liver failure, portal shunt animal models, and cellular models [140,141]. Increased levels of lactate and alanine were found in rats suffering from acute liver failure, possibly due to increased glycolysis or reduced flux through the TCA cycle, resulting in pyruvate accumulation [142]. However, in models of hepatic encephalopathy, changes in glucose metabolism are inconclusive [143]. In addition, abnormal lipid metabolism, especially an increase in short-chain fatty acids, is also an important feature in the pathogenesis of HE [144]. In conclusion, cerebral metabolic oxygen consumption and cerebral blood flow are drastically reduced in HE patients. The accompanying hypoxia and metabolic disturbances of sugar, water, and electrolytes can interfere with brain energy metabolism and exacerbate encephalopathy [144].

Recent studies have found that mitochondrial GDH plays an important role in the pathogenesis of HE through metabolic reorganization of the TCA cycle [130,145]. Within minutes after treatment of rat astrocytes or human brain astrocytomas with ammonium chloride, altered mitochondrial morphology and impaired oxidative phosphorylation were observed. Although basal mitochondrial respiration was only slightly affected in this scenario, spare respiratory capacity was progressively reduced according to increasing ammonia concentrations [130]. Targeted metabolic flux and isotope metabolomics analysis showed that ammonia was immobilized in glutamate within minutes after administration and gradually accumulated in aspartate and proline over time, suggesting the involvement of GDH, particularly human GDH2 (hGHD2), and downstream transaminases [130]. Inhibition of hGDH2 expression or the addition of glutamine and glutamate reduced ammonia-induced rapid damage to mitochondrial respiration [130]. Dadsetan et al. also found that in rat brains, ammonia was more immobilized in glutamate, alanine, and aspartate by GDH and transaminases when GS was inhibited [146].

### 2.8. Senescence

Astrocyte senescence is thought to have an important role in brain dysfunction in neurodegenerative diseases, with potential mechanisms including impaired growth factor signaling, disrupted synaptic glutamate homeostasis, and unstable synaptic contacts [147,148]. Recent studies have found that aging is also an important factor contributing to the pathogenesis of HE, especially in astrocytes [15,16,71,81,149,150]. In astrocytes, ammonia downregulates miR326-3p, targeting HO-1 and NOX4 through the glycosylation of proteins such as 3-phosphoglyceraldehyde dehydrogenase, resulting in elevated free ferrous iron and H_2_O_2_ inside the cells. Thus, ammonia leads to activation of p53 and transcription of the cell cycle repressor genes p21 and GADD45α, which in turn trigger senescence [16,71,97]. The upregulation of senescence markers p21, p53, and GADD45α was also found in brain specimens of HE patients [71]. This could explain the persistence of some HE symptoms after the subsidence of the acute overt hepatic encephalopathy onset [6,151]. Recent studies have also found that ammonia toxicity-induced downregulation of astrocyte arginine methyltransferase 1 (CARM1) can inhibit proliferation and lead to senescence in both in vitro and animal models of HE [114].

Osmotic stress can also activate the Src/ERK1/2/p38MAPK signaling pathway in hepatocytes and upregulate miR-141-3p, which targets cell cycle protein-dependent kinase 8 (Cdk8), thereby inhibiting hepatocyte proliferation and triggering senescence [152].

### 2.9. Central and Peripheral Inflammation

Microglia play a central role in brain inflammation because they are a powerful source of RNOS and inflammatory factors. Microglia secrete large amounts of pro-inflammatory cytokines after being activated by stimulation or adopting a reactive phenotype. Although activated microglia may be neuroprotective [153], reactive microglia are thought to be hallmarks of neuroinflammation as pro-inflammatory cytokines and NLRP3 inflammatory vesicles derived from them can trigger brain dysfunction [154,155,156,157]. In post-mortem brain samples from patients with hepatic encephalopathy, upregulations of anti-inflammatory M2 microglia markers were observed, while expression levels of pro-inflammatory cytokines IL-1β, IL-4, IL-10, IFN-γ, and TNF-α remained unchanged [17,90,158]. Thus, investigators hypothesized that microglia in hepatic encephalopathy are activated but not reactive, which may help protect patients from brain dysfunction [17,90,158]. Evidence of microglial activation has also been observed in ammonia-exposed microglia in vitro and in different animal models of hepatic encephalopathy [90,159,160,161,162], while consistent neuroinflammation was not always observed [51,156,159,160,163]. Recent studies have also found that the presence of astrocytes is crucial for the ability of ammonia to reduce LPS-induced activation and synthesis of pro-inflammatory cytokines [164]. Extracellular vesicles from animals with chronic hyperammonemia contain enriched TNF- α, which further induces neuroinflammation in these models of minimal hepatic encephalopathy [165]. Activation of the brain-derived neurotrophic factor (BDNF)/tyrosine kinase receptor B (TrkB) signaling pathway and regulation of GABAA receptors play an important role in the development of neuroinflammation [165,166,167]. In astrocytes, highly expressed and autocrine S100B can also stimulate the expression of vascular endothelial growth factor (VEGF), which in turn induces the activation of nuclear factor-κB (NF-κB), ultimately leading to oxidative stress and neuroinflammation [168]. 

In animals developing acute or chronic liver failure and hepatic encephalopathy, recent studies have also pointed out the crucial participation of peripheral inflammation and circulating cytokines in brain dysfunction [159,169]. The potential mechanisms may include cytokine-induced weakening of the blood-brain barrier [163,170]. Interestingly, clinical studies have revealed that changes in the gut microflora are strongly associated with the secretion of pro-inflammatory cytokines [171], and correspondingly, probiotics and fecal transplants can be effective in alleviating HE symptoms [172,173]. Rifaximin can also improve HE symptoms such as dyskinesia by regulating intestinal microflora, effectively reducing peripheral inflammation, decreasing immune cell infiltration, and restoring normal neurotransmission [159,174,175,176,177]. In addition to rifaximin, carvedilol, an adrenergic receptor antagonist, could also potently suppress NF-κB activity and expression of pro-inflammatory cytokines in the brains of mice suffering HE [78]. Golexanolone was also shown to be able to improve cognitive and motor function in a rat model of HE via decreasing peripheral inflammation and neuroinflammation, which are related to the TNFR1-glutaminase-GAT3 and TNFR1-CCL2-TrkB-KCC2 pathways [178]. Alternative medicine such as Babao Dan and electroacupuncture treatment were also shown to be able to reduce levels of pro-inflammatory cytokines in animal models of HE, showing the therapeutic potential of alternative medicine to treat HE [179,180].

Notably, inflammation is also closely linked to other mechanisms of hepatic encephalopathy. In astrocytes, inflammatory cytokines can also induce protein tyrosine nitration and astrocyte swelling [64,85,181]. Mitochondrial dysfunction can also stimulate the synthesis of inflammatory cytokines in various cells and is thought to play a key role in neurological and peripheral inflammation [182].

### 2.10. Other Cellular Pathogenesis Theories of Hepatic Encephalopathy

In the pathogenesis of hepatic encephalopathy, ammonia toxicity and manganese toxicity have synergistic effects in many aspects [183]. In addition to ammonia toxicity and manganese toxicity, the main proven theories are plasma amino acid imbalance and pseudoneurotransmitters [184,185]. The theory of the plasma amino acid imbalance suggests that hepatic encephalopathy is induced by a decrease in branched-chain amino acids and an increase in aromatic amino acids in the plasma due to liver damage. The pseudoneurotransmitter theory suggests that due to hepatic injury lesions or via the collateral circulation, phenylethylamine and p-hydroxyphenylethanolamine concentrations are elevated in the brain, which in turn leads to elevated phenylethanolamine and p-hydroxyphenylethanolamine concentrations. As phenylethanolamine and p-hydroxyphenylethanolamine are structurally similar to but much less potent than the normal neurotransmitters norepinephrine and dopamine, they compete with the normal neurotransmitters to promote brain dysfunction [184,185].

## 3. Conclusions

Research over the past decades has identified various molecular and cellular-level disorders in hepatic encephalopathy. These major advances and representative studies are summarized in Table 1. Still, the comprehensive and detailed pathogenesis of hepatic encephalopathy is not yet clear. Thus, to date, the vast majority of treatments for hepatic encephalopathy have focused on the elimination of precipitating factors, with a particular focus on ammonia reduction, but treatments that directly target pathophysiological processes in the brain are still lacking. 

Potential intervention strategies include counteracting oxidative/nitrosative stress and its sequelae in the brain, as well as modulating pathological oscillations. In recent years, additional potential HE therapeutic targets and strategies have been identified from the mechanistic study of brain cell damage, such as modulation of autophagy, mitochondrial function, senescence, and inflammation. 

A more thorough understanding of the hepatic encephalopathy pathophysiology will facilitate the emergence of more economical, efficient, and safe therapies to effectively reduce the morbidity and mortality of hepatic encephalopathy.

## Figures and Tables

**Table 1:** An overview of major molecular and cellular pathogenesis mechanisms of hepatic encephalopathy and their representative evidence.

Mechanisms	In Vitro Model		In Vivo Model		Patient/Post-Mortem Samples	
	Evidence Available	Reference	Evidence available	Reference	Evidence Available	Reference
Ammonia toxicity	√	[30,71,84,123]	√	[10,11,55,61,98]	√	[21,22]
Manganese toxicity	√	[39,40,41,42]	√	[45,46,47]	√	[35,36,37]
Astrocyte swelling and cerebral edema	√	[60,62,76,96]	√	[31,88]	√	[10,11]
Upregulation of AQ4 in the plasma membrane	√	[84,85]	√	[72]	N.A.	
Oxidative/Nitrosative stress	√	[16,51,52,53,54,57,58,60,61,62,64]	√	[10,11,51,52,53,55,56,59,61,63]	√	[10,11,16,17]
Protein tyrosine nitration	√	[52,61,62,64,96]	√	[52,59,61,64,106,107]	√	[11]
Protein phosphorylation	√	[61,79]	√	[160]	N.A.	
Protein glycosylation	√	[97,98]	√	[98]	N.A.	
Protein carbonylation	√	[99]	√	[100]	N.A.	
Protein ubiquitination	√	[99]	√	[100]	N.A.	
RNA oxidation	√	[51,53]	√	[51,53,59]	√	[11]
Alterations in gene expression/signaling pathways	√	[16,57,67,81,115,121]	√	[57,116,117]	√	[17,71,118,121]
Autophagy and lysosome damage	√	[123,127]	√	[123,124]	√	[123]
Mitochondrial bioenergetic alterations	√	[30,32]	√	[141,142]	√	[140]
Mitochondrial permeability transition	√	[30,32]	N.A.		N.A.	
Mitochondrial fragmentation and enlargement	√	[71,130,131]	√	[131]	N.A.	
Senescence	√	[16,71,81,149,150]	√	[114]	√	[16,71]
Activation of microgalia/inflammation	√	[164,168]	√	[90,159,160,161,162,168]	√	[17,90,158]

√: evidence available; N.A.: evidence not available yet.

## Data Availability

Not applicable.

## Abbreviations

αKG: α-ketoglutarate; AQ4: aquaporin-4; ATP: adenosine triphosphate; BDNF: brain-derived neurotrophic factor; CARM1: astrocyte arginine methyltransferase 1; Cdk8: cell cycle protein-dependent kinase 8; EGLN3: egl-9 family hypoxia-inducible factor 3; ERK: extracellular signal-regulated kinase; GABA: γ-aminobutyric acid; GDH: glutamate dehydrogenase; GGC: glutamine-glutamate cycle; GLAST: glutamate aspartate transporter protein; GLT-1: glutamate transporter protein 1; GLS: glutaminase; GS: glutamine synthetase; HE: hepatic encephalopathy; HO-1: heme oxygenase; iNOS: inducible nitric oxide synthase; JNK: c-Jun N-terminal kinase; LC3B: microtubule-associated protein 1A/1B light chain 3B; LPS: lipopolysaccharide; MRI: magnetic resonance; MRP4: multidrug resistance protein 4; MSO: methionine sulfoximine; MT: metallothionein; MTF: metal response element-binding transcription factor; NF-κB: nuclear factor-κB; NKCC1: sodium-potassium-chloride cotransport protein; NMDAR: N-methyl-d-aspartate receptor; nNOS: neuronal isozymes; Nrf2: nuclear factor-erythroid 2 related factor 2; NOS: nitric oxide synthase; NOX: NADPH oxidase; PBR: peripheral-type benzodiazepine receptor; PPAR: peroxisome proliferator-activated; RNOS: reactive nitrogen oxide species; ROS: reactive oxygen species; Slc38: solute carrier 38; SMIT: sodium-dependent inositol transporter protein; SP: specificity protein; TAUT: taurine transporter protein; TCA: tricarboxylic acid; TGR5: G-protein-coupled bile acid receptor; TNF-α: tumor necrosis factor α; TrkB: tyrosine kinase receptor B; VEGF: vascular endothelial growth factor; YY1: Yin Yang 1.
